# Ectopic expression of SARS-CoV-2 S and ORF-9B proteins alters metabolic profiles and impairs contractile function in cardiomyocytes

**DOI:** 10.3389/fcell.2023.1110271

**Published:** 2023-02-22

**Authors:** Peng Zhang, Yu Liu, Chunfeng Li, Lindsay D. Stine, Pei-Hui Wang, Matthew W. Turnbull, Haodi Wu, Qing Liu

**Affiliations:** ^1^ Department of Biological Sciences, Clemson University, Clemson, SC, United States; ^2^ Stanford Cardiovascular Institute, Stanford University School of Medicine, Stanford, CA, United States; ^3^ Institute for Immunity, Transplantation, and Infection, Stanford University, Stanford, CA, United States; ^4^ Southern Illinois University School of Medicine, Springfield, IL, United States; ^5^ Key Laboratory for Experimental Teratology of Ministry of Education and Advanced Medical Research Institute, Cheeloo College of Medicine, Shandong University, Jinan, China; ^6^ Department of Medicine, Division of Cardiology, Heart, Lung, Blood, and Vascular Medicine Institute, University of Pittsburgh, Pittsburgh, PA, United States; ^7^ Center for Human Genetics, Clemson University, Greenwood, SC, United States

**Keywords:** SARS-CoV-2, S, 9b, cardiomyocyte, metabolism

## Abstract

Coronavirus disease 2019 (COVID-19) is associated with adverse impacts in the cardiovascular system, but the mechanisms driving this response remain unclear. In this study, we conducted “pseudoviral infection” of SARS-CoV-2 subunits to evaluate their toxic effects in cardiomyocytes (CMs), that were derived from human induced pluripotent stem cells (hiPSCs). We found that the ectopic expression of S and ORF-9B subunits significantly impaired the contractile function and altered the metabolic profiles in human cardiomyocytes. Further mechanistic study has shown that the mitochondrial oxidative phosphorylation (OXPHOS), membrane potential, and ATP production were significantly decreased two days after the overexpression of S and ORF-9B subunits, while S subunits induced higher level of reactive oxygen species (ROS). Two weeks after overexpression, glycolysis was elevated in the ORF-9B group. Based on the transcriptomic analysis, both S and ORF-9B subunits dysregulated signaling pathways associated with metabolism and cardiomyopathy, including upregulated genes involved in HIF-signaling and downregulated genes involved in cholesterol biosynthetic processes. The ORF-9B subunit also enhanced glycolysis in the CMs. Our results collectively provide an insight into the molecular mechanisms underlying SARS-CoV-2 subunits-induced metabolic alterations and cardiac dysfunctions in the hearts of COVID-19 patients.

## Introduction

Coronavirus disease 2019 (COVID-19) is a potentially fatal respiratory disease caused by severe acute respiratory syndrome coronavirus 2 (SARS-CoV-2). There have been 635 million confirmed cases of COVID-19 globally as of 23 November 2022, including 6.6 million deaths reported the World Health Organization (WHO). COVID-19 related morbidity and mortality also exert a devastating impact on global public health and socio-economic development. The SARS-CoV-2 is a single-stranded RNA virus athat shows great and fast mutation potential (Hoffmann et al., 2020; Azevedo et al., 2021). Viral infection occurs through the binding the surface spike protein (i.e., S protein) with angiotensin-converting enzyme 2 (ACE2), which acts as the primary receptor for the virus. ACE2 is highly expressed in the lungs and also present in large amounts in the heart, causing cardiovascular complications *via* binding with the S protein of the virus (Hoffmann et al., 2020; Redondo et al., 2021; Farooq et al., 2022). However, the exact mechanisms underlying the cardiovascular complications induced by individual SARS-CoV-2 subunits remain largely unknown.

The symptoms of COVID-19 vary from asymptomatic, mild disease, to acute respiratory distress syndrome (ARDS). While SARS-CoV-2 infection causes mild upper respiratory disease or even asymptomatic symptoms in the majority of patients, others develop ARDS, which can be fatal (Clerkin et al., 2020; Wu and McGoogan, 2020). It is understood that the cause of the severe COVID-19 symptoms are partially due to the cytokine dysregulation and hyperinflammation in patients, and is triggered by impaired interferon responses (Arunachalam et al., 2020; Hadjadj et al., 2020). Moreover, pre-existing cardiovascular diseases and risk factors were highly correlated well with the severity of COVID-19. Although the respiratory system is the major target of SARS-CoV-2, infection also can damage other organs. The hospitalized patients with COVID-19 showed elevated cardiac troponin I levels, which indicates the cardiac damages after infection (Shi et al., 2020; Zhou et al., 2020), and patients with cardiovascular disease showed higher mortality (Ruan et al., 2020). This leads to the aggravation of chronic underlying cardiac pathologies as well as acute-onset of new cardiovascular complications, indicating that myocardial injury can be present in some hospitalized patients with SARS-CoV-2 infection (Clerkin et al., 2020; Wu and McGoogan, 2020).

Cardiomyocytes (CMs) derived from human stem cells provide a great model for mechanistic and toxicologic studies in the cardiovascular system. In our previous studies, we established a human stem cell-based and omics-centric approach, in which transcriptomes and epigenomes were interrogated to identify the transcriptional regulatory mechanisms subserving early CM differentiation and drug responses in the differentiated cells (Liu et al., 2017; Zhao et al., 2017). Human stem cells-derived CMs have been applied to evaluation of toxicities from cancer drugs (Sharma et al., 2015; Wang et al., 2019). More recently, multiple investigators have reported the transcriptomic and functional remodeling induced by SARS-CoV-2 infection in iPSC-derived CM models, and this improved our understanding of COVID-19 related cardiac risks by exploiting human-originated cellular models (Sharma et al., 2020; Yang et al., 2020; Yang et al., 2021). However, although it is known that SARS-CoV-2 overexpresses nucleocapsid proteins in host cells after transfection, it remains unclear as to the specific role of each one of them in inducing the toxicity and functional failure in the heart (Bailey et al., 2021). In the present study, we generated “pseudoviral infection” of SARS-CoV-2 subunits in the CMs derived from human induced pluripotent stem cells (hiPSCs), and evaluated cardiac functions and metabolic profiles. Genome-wide transcriptomics was implemented to understand mechanisms underlying SARS-CoV-2 subunits-induced adverse impacts on CMs.

## Materials and methods

### Lentiviral plasmids construct for SARS-CoV-2 subunits

The sequences of SARS-CoV-2 (Wuhan-Hu-1 strain, GenBank: NC_045512.2) was used as template to synthesize each structural gene (General Bio, China). 11 viral genes and constructed into the lentivirus vector separately. These genes included: Structural genes (spike [S], membrane [M], Envelope [E], and Nucleocapsid [N], and open reading frames (ORF)-3, ORF-6, ORF-7A, ORF-7B, ORF-8, ORF-9A, ORF-9B (Zhang and Holmes, 2020). The viral genes were firstly cloned into pcDNA6B-Flag vector as described previously (Zhang et al., 2021), and then they were subcloned into a lentivirus plasmid pCDH-CMV-MCS-EF1α-copGFP (System Biosciences, #CD511B-1, Palo Alto, CA, United States) with standard molecular cloning methods. The vector only contains GFP-reporter was used as a control. Among them, the DNA sequences of S and ORF-3 were codon optimized to ensure a high expression level in human cells.

### Cell culture and cardiomyocyte differentiation

The hiPSC line was adopted in this study after obtaining them from the Stanford Cardiovascular Institute (SCVI) Biobank, Stanford University. It was generated thorough reprograming of peripheral blood mononuclear cells (PBMCs) from an anonymous healthy individual with Sendai virus. The pluripotent cell lines were grown in Matrigel (Corning, CA)-coated 12-well plates in Essential 8™ Medium (Thermo Fisher Scientific, MA) at 37°C in 5% CO2 in compressed air and high humidity. Cardiomyocyte differentiation was conducted using a monolayer differentiation chemically defined method (Burridge et al., 2014). Briefly, iPSCs were kept in culture until 80%–90% confluence. For the differentiation, iPSCs were treated with 6 µM CHIR99021 in RPMI + B27 (minus insulin) for 2 days, fresh RPMI + B27 (minus insulin) for 1 day, followed by 5 µM IWR-1 treatment for 2 days, and then fresh RPMI + B27 (minus insulin) for another 2 days. Afterward, the cells will be supplied with fresh RPMI + B27 every other day. Beating cardiomyocytes will normally appear after 9–10 days, and the cells can be further treated with glucose free RPMI + B27 for 2–3 rounds.

### Lentivirus preparation and transfection of cardiomyocytes

HEK293T (ATTC, Cat# CRL-3216) cells were kept in 6-well plates with Dulbecco’s Modified Eagle Medium (DMEM, Gibco) supplemented with 10% fetal bovine serum. Packaging plasmids (pVSVg and psPAX2), pCDH containing SARS-CoV-2 subunits, Opti-MEM (Thermo Fisher Scientific), and X-tremeGENE 9 DNA transfection reagent (Sigma-Aldrich) were combined to transfect HEK293T cells according to the manufacturer’s instructions. Medium supernatants containing virus particles were filtered through a 0.45-μM filter and further concentrated using a Lenti-x concentrator (Takara Bio) according to the manufacturer’s protocol. 2 μg/ml of polybrene was used for transfection of differentiated cardiomyocytes, and puromycin was used to select the transduced cells. Since the plasmids can express GFPs, successful transfection and expression of each unit were determined by evaluating the GFP with a Nikon Ti2-E fluorescence microscope (Supplementary Figure S1).

### Metabolic profiling by seahorse experiments

We used XF Cell Mito Stress Test and XF Glycolytic Rate Assay kit to measure the oxygen consumption rate (OCR) for the mitochondrial respiratory activity and proton efflux rate (PER) for the glycolytic levels in the cardiomyocytes, by using a Seahorse XFe96 Extracellular Flux Analyzer (Agilent, CA). Cells (45,000) were plated into an Xfe96 cell culture microplate (Agilent) containing RPMI/B27 supplemented with 10% FBS and 10 μM ROCK inhibitor. After 48 h to allow recovery, we conducted the metabolic profiling using the XFe96 Seahorse analyzer with two kits according to the manufacture’s manual. Briefly, 1 day prior to the experiment, the Xfe96 sensor cartridges were hydrated in XF calibrator solution and incubated overnight at 37°C in a non-CO_2_ incubator. 1 hour prior to the experiment, the cells were incubated at 37°C (non-CO_2_) in 200 μl of Seahorse assay medium, containing XF base medium supplemented 1 mM pyruvate, 2 mM glutamine, and 10 mM glucose (pH 7.4). OCR was measured with sequential injections of 2 μM oligomycin, 2 μM FCCP and each 0.5 μM of rotenone/antimycin A. PER was measured with sequential injections of 0.5 μM of rotenone/antimycin A and 50 mM of 2-deoxy-D-glucose (2-DG). Data were normalized by fluorescence of cell viability using PrestoBlue reagent (Thermo Fisher).

### RNA-isolation

Total RNA was extracted from the same number of cells among each group using QIAzol lysis reagent (Qiagen), and RNA was then subjected to Dnase I digestion and purified using a miRNeasy Mini Kit (Qiagen) according to the manufacturer’s instructions. RNA integrity was assessed with a NanoDrop, and only samples with a ratio of 260/280 between 2.0—2.1 were subsequently used for ribosome depletion.

### RNA-sequencing and analysis

The library preparation and RNA-sequencing were performed by Novogene Corporation Inc., (Sacramento, CA). The RNA-seq libraries were constructed using NEBNext UltraTM II RNA Library Prep Kit for Illumina and were sequenced by Novaseq 6,000 PE150 system. Raw reads of FASTQ format were firstly processed through fastp, and clean data (clean reads) were obtained by removing reads containing adapter and poly-N sequences and reads with low quality from raw data. All the downstream analyses were based on the clean data with high quality. The raw RNA-seq raw data were trimmed to remove the adapter sequences (GAT​CGG​AAG​AGC​ACA​CGT​CTG​AAC​TCC​AGT​CAC​GGT​CTA​CTA​TCT​CGT​ATG​CCG​TCT​TCT​GCT​TG and AGA​TCG​GAA​GAG​CGT​CGT​GTA​GGG​AAA​GAG​TGT​AGA​TCT​CGG -TGGTCGCCGTATCATT) with command-line tool cutadapt (1.8.1). Then the trimmed files were aligned with Tophat (version 2.0.9) to GRCh37/hg19 *Homo sapiens* reference genome. The human gene symbols and their raw counts were calculated using the HTSeq (version 0.6.1p1) package in Python with the hg19 *Homo sapiens* gtf file. Differential gene-expression analysis was performed using the edgeR package in R, and the normalization was performed using a trimmed mean of M-values (TMM) method across all samples. The Gene Ontology (GO) enrichment analysis was performed using on-line tools DAVID (version 6.8) (https://david.ncifcrf.gov/summary.jsp) and the Gene Ontology Resource (http://geneontology.org). The Gene Ontology (GO) enrichment analysis of differentially expressed genes was performed using DAVID (https://david.ncifcrf.gov).

### Western blot analysis

The cells were harvested in RIPA lysis buffer (EMD Millipore, CA) contain one tablet of Pierce™ protease and phosphatase inhibitor (Thermo Fisher Scientific), and the proteins were purified using a Branson Digital Sonifier homogenizer (Branson Ultrasonics, CT). 20 μg of protein from each sample was separated on NuPAGE 4–12% Bis-Tris protein gels (Thermo Fisher Scientific) and transferred to nitrocellulose membranes (Thermo Fisher Scientific). The protein-bound membranes were blocked with 5% of blotting-grade blocker (Bio-Rad) in PBST for 1 h at room temperature and incubated with a primary antibody (1:1,000 dilution) in 5% of blotting-grade blocker in PBST overnight at 4°C. After washing with PBST buffer, the membranes were incubated with horseradish peroxidase (HRP)-conjugated-secondary antibody for 1 h at room temperature. The membranes were developed with SuperSignal West Femto Maximum Sensitivity Substrate (Thermo Fisher Scientific) and exposed on a ChemiDoc Touch imaging system (Bio-Rad) for imaging. The primary antibody used in this study was total OXPHOS human WB antibody cocktail (Abcam, ab110411). The secondary antibodies was HRP-conjugated-goat anti-mouse IgG (SouthernBiotech, 1030-05).

### Functional analysis of iPSC-derived cardiomyocyte

For contractile function analysis, CMs were seeded on 96 well plates at a density of 100 k cells per well. Cells will start to beat after 2–3 days of recovery, and were further matured until day 30 after differentiation before functional measurement. The contractile function of beating cardiomyocytes was analyzed using a Sony SI18000 cell motion imaging system. Briefly, high-resolution and high-frame rate contractile videos (1,024*1,024 at 75fps) were recorded, and the key contractility parameters, such as contractile velocity (μm/s), relaxation velocity (μm/s), percentile of beating area, and contraction/relaxation durations (s) were calculated according to the pixel displacement between video frames. To better evaluate the effect of COVID-19 protein overexpression in the CMs, the treatment duration was set to 1 week, and we measured the contractile function both before and after lentivirus infection.

### Mitochondrial DNA dynamics analysis

DNA from cardiomyocytes was isolated using AllPrep DNA/RNA Mini kit (Qiagen), and the human mitochondrial to nuclear DNA ratio kit (Takara) was used to determine mitochondrial DNA content. Two separate primer pairs were used to generate nuclear-mitochondrial DNA content ratios. SLCO2B1 and SERPINA1 were used as nuclear genes, while ND1 and ND5 were used as mitochondrial genes. Two genes for both nuclear and mitochondrial DNA were used as an average to prevent outliers. Average of ratio between mitochondrial genes and nuclear genes was used to determine the mitochondrial DNA content of each sample.

### High content imaging

ATP production, mitochondrial membrane potential, and gross mitochondrial size of differentiated cardiomyocytes were evaluated using an imaging-based multi-parametric analysis strategy (i.e., high content imaging). Differentiating cardiomyocytes were re-plated into optical 96-well plates, and cells were labeled with the following fluorescent dyes: BioTracker ATP-red live cell dye (Sigma,SCT045), tetramethylrhodamine, methyl ester (Thermo Fisher Scientific, I34361), and CellROX Orange Reagent (Thermo Fisher Scientific, C10443). The fluorescence intensities or areas were quantified using a Cytation one cell imaging multi-Mode reader with Gen5 Image Prime software (BioTek).

### Statistical analysis

We conducted statistical analysis using GraphPad Prism 8.4 (GraphPad Software, Inc., San Diego, CA). Non-parametric t-test was used to compare data between two groups and one-way or two-way ANOVA followed by Tukey’s test were used to compare data of multiple groups wherever appropriate. Data are reported as means ± standard error of the mean (SEM).

## Results

### Inducing expression of SARS-CoV-2 subunits altered cardiac functions in cardiomyocytes

**FIGURE 1:**
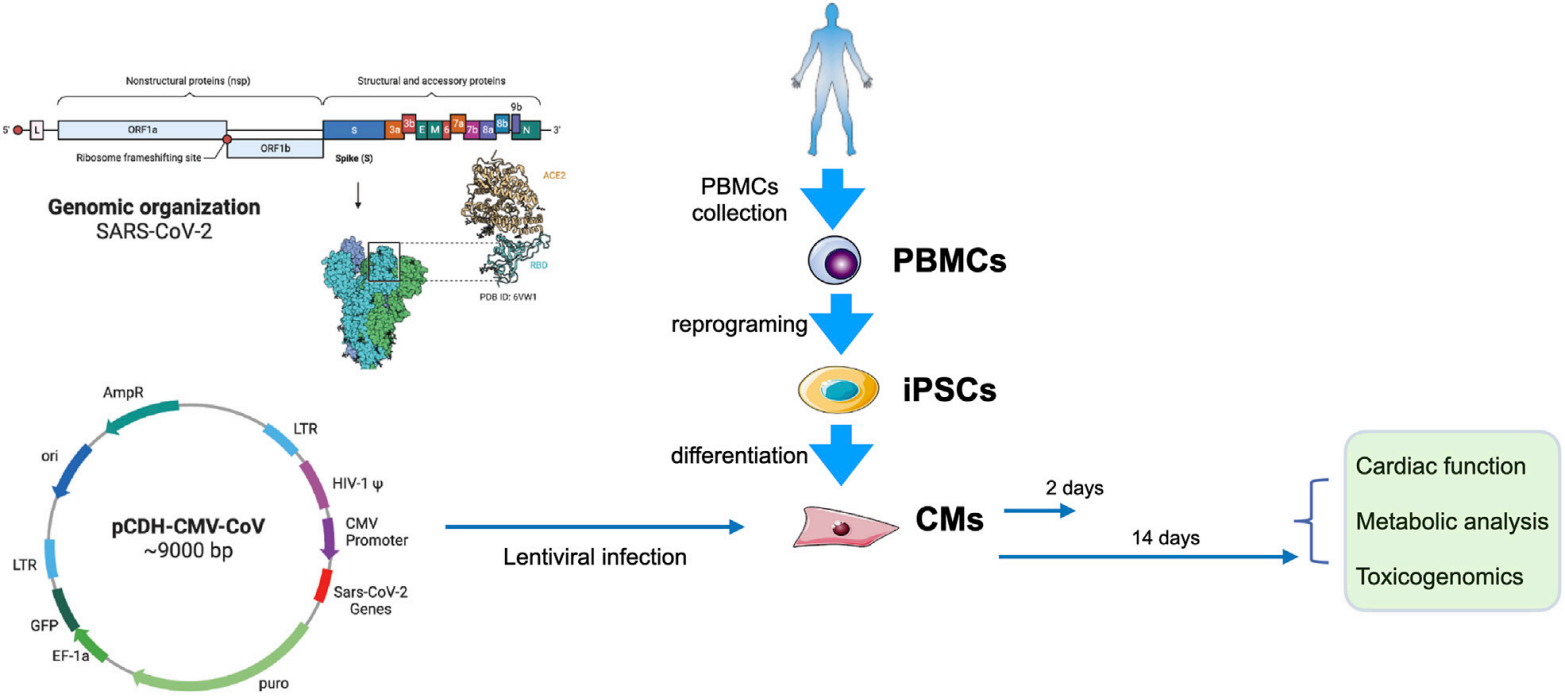
Experimental design. Left, the genomic organization of SARS-CoV-2 (up) and the map of pCDH-CMV-CoV (down). Right, PBMCs from a health donor were collected and reprogrammed into iPSCs, and then were differentiated into CMs. The CMs were transduced with lentivirus carrying SARS-CoV-2 genes on day 30. Cells we collected for functional, metabolic, and toxicogenomic analyses 2 days (day 32) or 2 weeks (day 45) after transfection.

**FIGURE 2:**
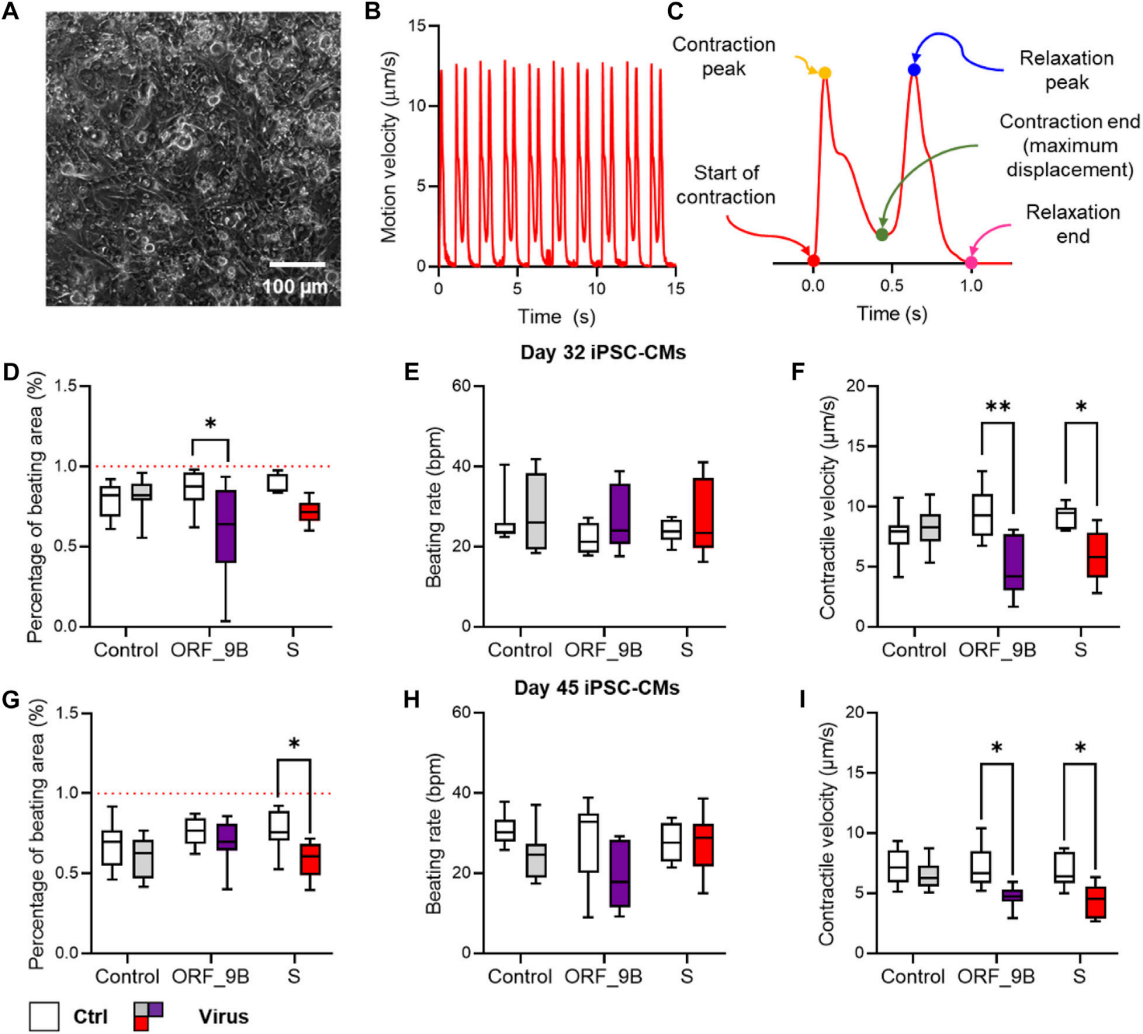
Screening of the impact of SARS-CoV-2 subunits in CMs with contractility assay. **(A)** Phase contrast image of monolayer CMs plated for contractility assay. **(B)** Typical trace of the average displacement velocity generated by frame-to-frame displacement analysis. **(C)** Averaged motion peaks showing the start, peak, and end of contraction and relaxation, allowing detailed analysis of contractile and relaxation velocity and duration. **(D–F)** Functional measurement of the percentage of beating area **(D)**, beating rate **(E)** and contractile velocity **(F)** in CMs before (control) and after (virus) over-expression of COVID proteins by lentiviral vectors. **(G–I)** Functional measurement of the percentage of beating area **(G)**, beating rate **(H)** and contractile velocity **(I)** in CMs on day 30 before (control) and after (virus) overexpression of COVID proteins by lentivirus vectors. N > 8 wells of CMs from at least two independent experiments in each group. * and **: *p* < 0.05 and *p* < 0.01 vs. non-virus controls from the same group of COVID-19 experiment in two way-ANNOVA followed by Sidak’s multiple comparison.

Based on the genomic structural of SARS-CoV-2 (Wuhan-1 strain), we evaluated the role of the 11 SARS-CoV-2 proteins in human cardiomyocytes. These proteins were transfected into the differentiated CMs on day 30. 2 days (day 32) and 2 weeks (day 45) after transfection the CMs were used for the analyses (Figure 1). The contractile function of the monolayer CMs were then calculated using a traction force microscopy recording platform (Figure 2A). Our results showed that the over-expression of two of the SARS-CoV-2 proteins (ORF-9B and the spike protein S) were significantly related to the reduced contractile function in CMs, out of all the SARS-CoV-2 proteins tested (Figures 2B, C). Specifically, overexpression of ORF-9B and S induced significantly reduce in the beating area (%BA) and the contractile velocity, while no changes in the beating rate was detected. The functional changes result from ORF-9B and S protein overexpression were consistent in both CMs on day 32 and day 45, indicating the potential roles of them in the regulation of contractile function. The overexpression of other SARS-CoV-2 proteins generally induced a varied result: although some of them induced slightly functional changes at certain timepoints and to certain parameters, yet their impact on the contractile function was still not conclusive (Supplementary Table S1). Thus, we herein focused specifically on ORF-9B and S and their regulatory roles in the subsequent studies.

### Expression of SARS-CoV-2 subunits inhibited mitochondrial OXPHOS but enhanced glycolysis

**FIGURE 3:**
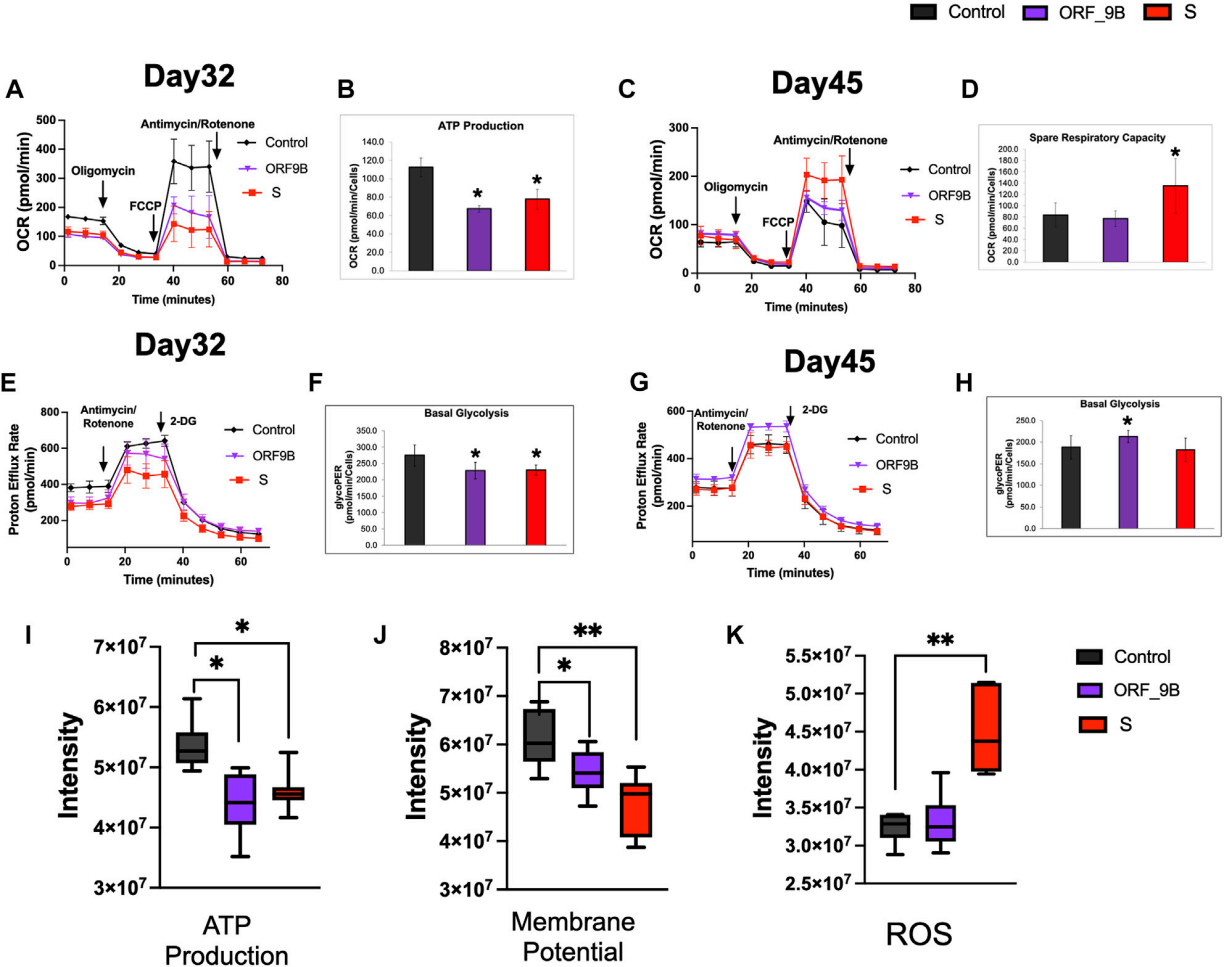
Evaluation of the effects of SARS-CoV-2 subunits on mitochondrial functions and glycolysis. **(A, B)** Evaluation of mitochondrial oxygen consumption rate (OCR) in CMs on day 32 after overexpression of S and ORF-9B subunits for two days. **(C, D)** Evaluation of mitochondrial oxygen consumption rate (OCR) in CMs on day 46 after overexpression of the S and ORF-9B subunits for two weeks. **(E, F)** Evaluation of glycolysis by measuring proton efflux rate (PER) in CMs on day 32 after overexpression of the S and ORF-9B subunits for 2 days. **(G, H)** Evaluation of glycolysis by measuring PER in CMs on day 45 after overexpression of S and ORF-9B subunits for 2 weeks. The data were normalized to cell numbers. **(H–K)** Mitochondrial ATP production **(I)**, mitochondrial membrane potential **(J)**, and reactive oxygen species (ROS) level in CMs were measured with a BioTek Cytation 1. Data are reported as means ± standard error of the mean (SEM). *p*< 0.05: **, *p* < 0.01.

Differentiated CMs principally use mitochondrial OXPHOS to support their large ATP demands; and we therefore examined mitochondrial OXPHOS by measuring oxygen consumption rate (OCR) using a Seahorse XFe96 Extracellular Flux Analyzer. We observed that short-term (2 days) induction of S and ORF-9B subunits caused diminutions in mitochondrial respiratory activity, including basal OCR, maximal and spare respiration, spare respiratory capacity, and ATP production (Figures 3A, B; Supplementary Figure S2), demonstrating an acute impairment on mitochondrial OXPHOS from S and ORF-9B proteins. Intriguingly, no significant differences in mitochondrial OXPHOS were observed between control and ORF-9B group after two weeks, except for higher maximal and spare respiration in S group (Figures 3C, D; Supplementary Figure S3); this suggests that CMs may change their metabolic profiles to adapted to infections after longer-term exposure to S and ORF-9B proteins. We then evaluated the glycolytic levels by measuring proton efflux rate (PER), and we found that both S and ORF-9B proteins caused slight reductions in basal glycolytic levels (Figures 3E, F, Supplementary Figure S4); however, inducing ORF-9B expression for two weeks evaluated basal and compensatory glycolysis (Figures 3G, H; Supplementary Figure S5). Short-term exposure to S or ORF-9B subunits was associated with attenuated ATP production and mitochondrial membrane potential (Figures 3I, J), and S protein was found to induce higher levels of reactive oxygen species (ROS) (Figure 3K). In addition, no significant alternations in mitochondrial DNA contents (mtDNA) or mitochondrial complexes were noted (Supplementary Figure S6). Collectively, these results suggested that SARS-CoV-2 subunits (i.e., S and ORF-9B) altered metabolic profiles without changing mitochondrial biogenesis.

### Transcriptomic analysis reveals a metabolic remodeling mechanism in CMs by inducing ORF-9B protein expression

**FIGURE 4:**
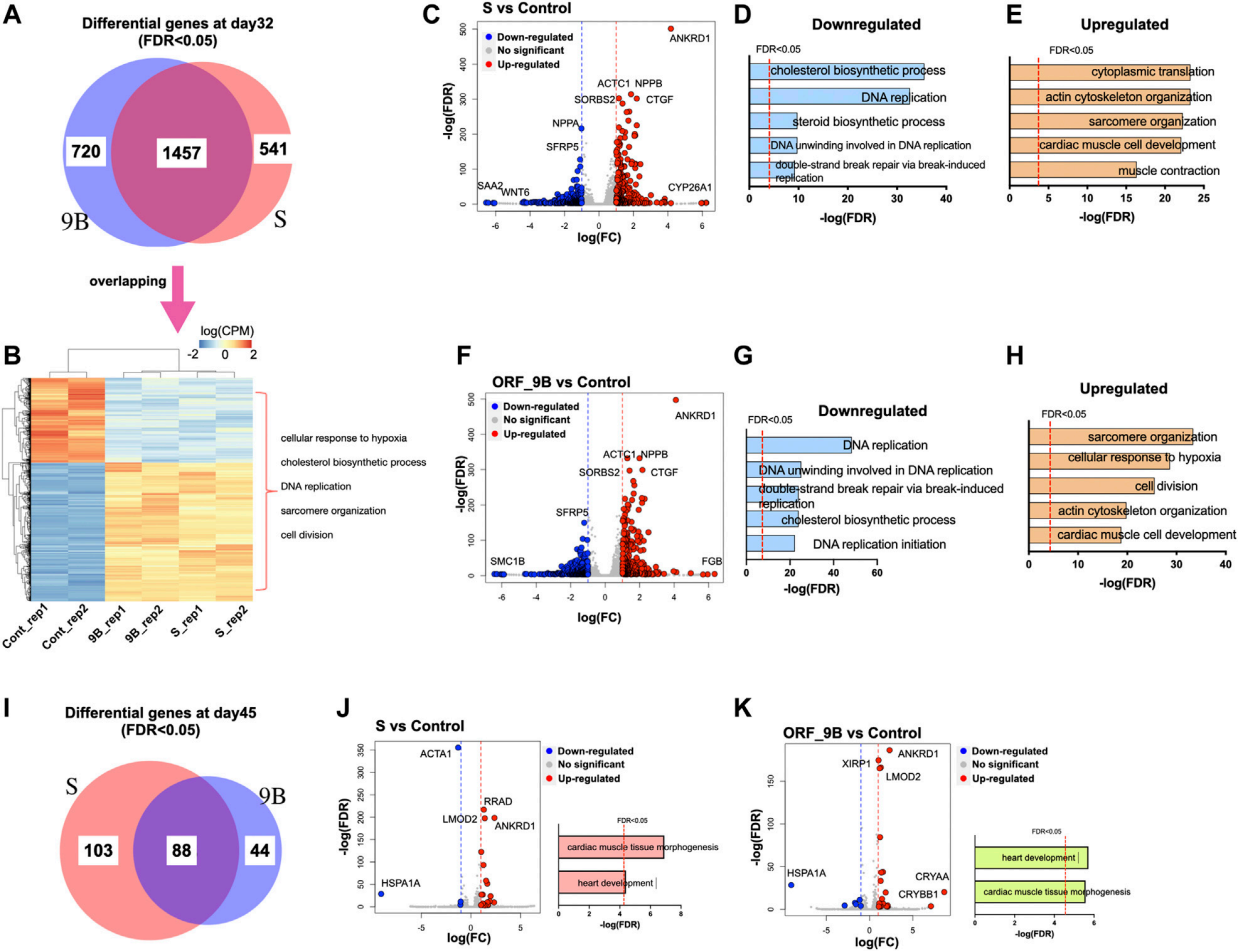
Transcriptomic analysis of CMs after overexpression of SARS-CoV-2 subunits. **(A)** The Venn diagram exhibits the statistically differential genes in CMs after overexpression ORF-9B or S (FDR < 0.05) day day32. **(B)** The heatmap exhibits the expression of overlapping differentially expressed genes in the ORF-9B and S group, compared to that in control. Each group had biological replicates. The representative enriched GO terms (FDR<0.05) are shown on the right side. **(C)** The scatter plot shows dysregulated genes in the S group compared to that in control (FDR < 0.05 with log[FC] larger than 1). Red, upregulated genes; blue, down-regulated genes. **(D, E)** The top enriched GO terms (FDR < 0.05) of the downregulated genes **(D)** and the upregulated genes **(E)** in the S group. **(F)** The scatter plot shows dysregulated genes in the ORF-9B group compared to that in control (FDR < 0.05 with log[FC] larger than 1). Red, upregulated genes; blue, down-regulated genes. **(G, H)** The top enriched GO terms (FDR < 0.05) of the downregulated genes **(G)** and upregulated genes **(H)** in the ORF-9B group. **(I)** The Venn diagram exhibits the statistically differential genes in CMs after overexpression ORF-9B or S (FDR < 0.05) day day45. **(J–K)** Left, the scatter plot shows dysregulated genes in the S group **(J)** and ORF-9B group **(K)** compared to that in control; right, the top enriched GO terms (FDR < 0.05) of the dysregulated genes of each group.

To elucidate the changes in gene expression during CM differentiation due to exposure to S and ORF_9B proteins, we performed genome-wide transcriptomic analysis of differentiated CMs using RNA-sequencing (RNA-seq) experiments. Based on the differential gene lists (FDR<0.05; Supplementary Tables S1, S2), we found that 1998 and 2,177 genes were dysregulated by S protein and 9B protein, respectively; and that over 50% were shared in common (Figure 4A). The enriched Gene Ontology (GO) terms of the commonly dysregulated genes were associated with “cellular response to hypoxia,” “cholesterol biosynthetic process,” “sarcomere structure,” etc., (Figure 4B), suggesting that both proteins dysregulated cardiac functions and metabolic process in CMs. The upregulated genes between S and ORF_9B groups showed a high similarity, including ankyrin repeat domain 1 (*ANKRD1*), actin alpha cardiac muscle 1 (*ACTC1*), connective tissue growth factor (*CTGF*), natriuretic peptide B (*NPPB*), and sorbin and SH3 domain-containing protein 2 (*SORBS2*) (Figures 4C–F). Cytochrome P450 family 26 subfamily A member 1 (*CYP26A1*) and fibrinogen beta chain (*FGB*) also revealed the highest fold changes in the S group and ORF-9B group, respectively (Figures 4C–F). However, downregulated genes showed slight differences between the two groups. For instance, serum amyloid A2 (*SAA2*) and WNT family member 6 (*WNT6*) were the two downregulated genes with the highest fold changes in the S group, compared to structural maintenance of chromosomes 1B (*SMC1B*) in the ORF-9B group. Moreover, dysregulated genes in both S and ORF-9B groups shared similar significantly enriched (FDR<0.05) GO terms: the down-regulated genes were associated with “DNA replication” and “cholesterol biosynthetic process” (Figures 4D–G); and the up-regulated genes are associated with “sarcomere organization,” “cardiac muscle cell development,” and “actin cytoskeleton organization” (Figures 4E–H).

After 2 weeks (i.e., on day 45), fewer dysregulated genes (FDR<0.05) in both the S and ORF-9B groups were uncovered in the CMs relative to day 32, and they were associated with “cardiac muscle tissue morphogenesis” and “heart development” (Figures 4I–K). Heat shock protein (Hsp70) family member 1A (*HSP1A1*) was the downregulated genes with the highest fold-change between the two groups; and crystallin alpha A (*CRYAA*) and crystallin beta B1 (*CRYBB1*) were the upregulated genes with the highest fold-changes only found in the ORF-9B group (Figures 4J–K).

**FIGURE 5:**
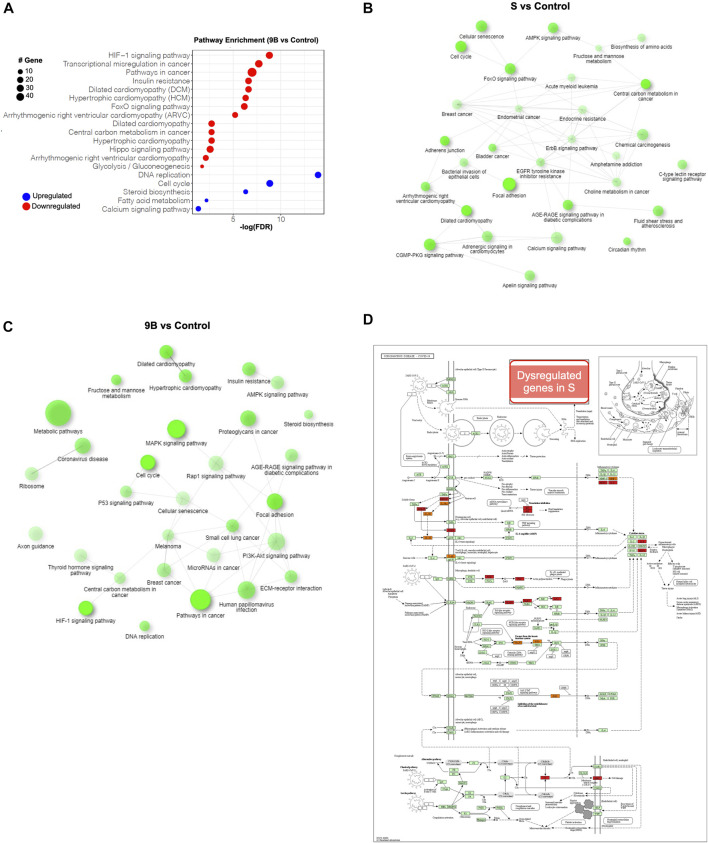
Pathway analysis of the differential genes in CMs after overexpression of SARS-CoV-2 subunits. **(A)** KEGG analysis of dysregulated genes in CMs of ORF-9B group. *Y*-axis shows statistically enriched (FDR < 0.05) KEGG pathways ranked by FDR. The red dots represent upregulated genes, and the blue dots represent downregulated genes. The size of the dot represents number sizes of dysregulated genes. **(B, C)** Interactive KEGG pathways (FDR < 0.05) from dysregulated genes from the ORF-9B group **(B)** and S groups **(C)**. **(D)** The KEGG map illustrates the dysregulated genes from S group that are in volved in the “coronavirus disease” pathway. Red, upregulated genes; blue, downregulated genes.

Based on the Kyoto Encyclopedia of Genes and Genomes (KEGG) analysis, we demonstrated that, “HIF-1 signaling pathway” was the most significantly upregulated pathway in both the S and ORF-9B groups. The upregulated genes in both groups are known to be involved in both cardiac diseases (e.g., “dilated cardiomyopathy,” “hypertrophic cardiomyopathy,” and “arrhythmogenic right ventricular cardiomyopathy”) and metabolic process (e.g., “FoxO signaling pathway,” “insulin resistance”); and the downregulated genes are involved in “steroid biosynthesis,” “fatty acid metabolism,” and “calcium signaling pathway” (Figures 5A–C; Supplementary Figure 7A). This demonstrated that the two SARS-CoV-2 subunits disrupted both cardiac metabolic processes and calcium handling properties in CMs. Most importantly, we also observed that upregulated genes in the ORF-9B group were involved in “glycolysis/gluconeogenesis” pathway, strongly supporting our glycolytic analysis in Figure 3 that shows elevated glycolytic levels in the CMs after induction of ORF-9B. In addition, the dysregulated genes in the S group were involved in “coronavirus disease,” suggesting a complex interaction among various signaling pathways during between coronavirus infection (Figures 5C, D).

## Discussion

In this study, we evaluated the effects of the subunits of coronavirus SARS-CoV-2 on CMs derived from human iPSCs. The S and ORF-9B subunits caused cardiac dysfunctions and metabolic alterations after long-term infection. Based on our toxicogenomic analysis, we ascertained that both S and ORF-9B subunits dysregulated several pathways involved in metabolism and cardiomyopathy, and ORF-9B subunit also enhanced the glycolysis, leading to metabolic remodeling in the infected CMs.

Cardiometabolic management is critical to maintaining normal cardiac function and health, and requires high energy-production (i.e., ATP) demands. Mature CMs mainly use mitochondrial OXPHOS to generate ATPs, while metabolic reprograming (or shifting) between glycolysis and mitochondrial OXPHOS is notable in cardiomyopathy (e.g., heart failure) patients (Lai et al., 2014; Rosano and Vitale, 2018). In addition, the relationship between the immune system and metabolism is highly linked to chronic metabolic diseases, such as diabetes and cardiovascular disease. Thus, disruption of metabolic homeostasis typically causes systemic inflammatory responses (Hotamisligil, 2017; Zmora et al., 2017), and this phenomenon may then explain why patients with diabetes and cardiovascular disease showed higher mortality from SARS-CoV-2 infection, presenting serve inflammatory syndromes (such as cytokines storm) (Drucker, 2021; Zhou et al., 2021). Some studies also reported that SARS-CoV-2 infection caused metabolism reprograming of various nutrients, such as glucose, fatty acid, cholesterol, and glutamine (Thomas et al., 2020; Caterino et al., 2021; Krishnan et al., 2021).

A study by Zhu et al. (Zhu et al., 2022) showed that expressing SARS-CoV-2 Nsp6 in *Drosophila* heart, leading to interaction with the host MGA/MAX complex (MGA, PCGF6, and TFDP1), ultimately causing metabplic swift to glycolysis. In addition, SARS-CoV-2 can manipulate mitochondrial functions of the host cells, probably by releasing ORFs proteins (such as ORF-9b) that can be localized into the host mitochondria (Singh et al., 2020)*.* In our study, both ORF-9B and S reduced mitochondrial OXPHOS levels and ORF-9B elevated glycolysis in CMs, demonstrating a smiliar metabolic reprogramming results from SARS-CoV-2 subunits in our human stem-cell-based system. In addition, S protein augmented the mitochondrial ROS levels (Figure 3K), and both ORF-9B and S proteins upregulated the HIF-1 signaling pathway (Figure 5A; Supplementary Figure 7B) and genes involved in “cellular response to hypoxia” (Figure 4H), suggesting a potential pathway: SARS-CoV-2 subunits induce hypoxia so as activate HIF-1 signaling, and then elevate glycolysis (Codo et al., 2020). This mechanism is observed in monocytes after SARS-CoV-2 infection, and is accompanied by increased ROS levels in mitochondria, which then activates HIF-1 signaling and glycolysis and eventually leads to cytokine storms (Codo et al., 2020). Our study suggests a similar mechanism in CMs from SARS-CoV-2 infection. In addition, cholesterol homeostasis is key to viral infection, and decreased HDL cholesterol levels and higher triglycerides have been demonstrated with SARS-CoV-2 infection (Hu et al., 2020; Wei et al., 2020; Masana et al., 2021). It is likely that SARS-CoV-2 infection induces the activation of sterol-regulatory element-binding protein 2 (SREBP-2), leading to disrupted cholesterol biosynthesis (Dai et al., 2022) or liver damages and lowered lipid metabolism (Kočar et al., 2021). In our study, both ORF-9B and S downregulated genes that are involved in “cholesterol biosynthetic process” suggesting alternations in lipid metabolism in the heart subsequent to infection.

Some of the significantly dysregulated genes from our study showed similarity to the previous clinical reports. For instance, higher levels of fibrinogen and CTGF were found in COVID-19 patients, and they serve as indictors for coagulation, fibrinolysis, and lung injury (Long et al., 2021; Sur et al., 2021; Laloglu and Alay, 2022), We discerned that these genes were also upregulated in CMs after infection, suggesting a similar pathologic progression in the CMs. In addition to these non-cardiac genes, SARS-CoV-2 subunits caused aberrant expression of cardiac genes in this study. From enriched GO terms of the upregulated genes in Figure 4, we hypothesize that ORF-9B and S subunits can alter the transcriptional regulation in cardiac gene programs. Additional mechanisms underlying transcriptional dysregulation need be investigated using other technologies, such as ATAC-seq, which we previously conducted in other toxicologic research (Liu et al., 2018). In addition to these findings, in this study we applied an *in-vitro* system to understanding of infectious disease *in vivo*. This stem-cell-based system provides huge advances in our studies on cardiovascular system of the COVID-19 patients, while it also has some limitations, such as immaturity of the differentiated cardiomyocytes compared to that mature heart *in vivo* (Lundy et al., 2013; Wu et al., 2015); and a lack of spatial and cellular heterogeneity in the monolayer model compared to that of heart. Thus, the mouse or 3D-cardiac organoids can offer an important complementary solution in the future studies.

## Data Availability

The RNA-seq data generated for this work have been deposited in the NCBI Gene Expression Omnibus, and they are accessible numbers are GSE202869.
