# Supplementary material for: Ectopic expression of SARS-CoV-2 S and ORF-9B proteins alters metabolic profiles and impairs contractile function in cardiomyocytes
Source: Front Cell Dev Biol. 2023 Feb 22;11:1110271. doi: 10.3389/fcell.2023.1110271 (PMC9994814; doi:10.3389/fcell.2023.1110271)
Supplement: Supplementary file 3 [file Table1.docx]

| **Readouts** | **% of beating area** | | **Contractile velocity (µm/s)** | | **Beating rate (bpm)** | |
| --- | --- | --- | --- | --- | --- | --- |
| **Treatment** | **Ctrl** | **Virus** | **Ctrl** | **Virus** | **Ctrl** | **Virus** |
| **Groups** | **Day 32 iPSC-CM data** | | | | | |
| **Ctrl** | 0.76 ± 0.03 | 0.69 ± 0.03 | 7.63 ± 0.45 | 6.84 ± 0.47 | 22.32 ± 0.45 | 28.65 ± 1.6** |
| **E** | 0.78 ± 0.04 | 0.86 ± 0.04 | 8.82 ± 0.78 | 8.98 ± 0.75 | 25.73 ± 1.59 | 34.26 ± 3.53* |
| **M** | 0.72 ± 0.06 | 0.74 ± 0.05 | 8.37 ± 0.97 | 7.4 ± 0.72 | 20.9 ± 0.33 | 27.28 ± 1.16 |
| **N** | 0.75 ± 0.04 | 0.74 ± 0.05 | 7.98 ± 0.68 | 6.47 ± 0.7 | 20.45 ± 1.34 | 24.58 ± 1.1 |
| **ORF-3** | 0.62 ± 0.06 | 0.69 ± 0.06 | 6.6 ± 0.37 | 6.44 ± 0.44 | 21.93 ± 0.63 | 24.95 ± 2.58 |
| **ORF-6** | 0.63 ± 0.07 | 0.67 ± 0.07 | 8.03 ± 0.99 | 6.36 ± 0.58 | 21.86 ± 1.12 | 23.15 ± 1.96 |
| **ORF-7A** | 0.66 ± 0.08 | 0.71 ± 0.07 | 7.71 ± 1.5 | 7.13 ± 0.73 | 17.23 ± 2.48 | 24.08 ± 0.9 |
| **ORF-7B** | 0.72 ± 0.06 | 0.82 ± 0.04 | 8.51 ± 0.7 | 8.68 ± 0.69 | 23.28 ± 0.52 | 25.79 ± 1.44 |
| **ORF-8** | 0.65 ± 0.06 | 0.69 ± 0.06 | 7.68 ± 0.43 | 7.34 ± 0.47 | 24.34 ± 0.96 | 24.96 ± 2.94 |
| **ORF-9A** | 0.78 ± 0.06 | 0.8 ± 0.07 | 9.71 ± 0.8 | 8.9 ± 1 | 24.09 ± 0.77 | 26.25 ± 1.07 |
| **ORF-9B** | 0.86 ± 0.04 | 0.6 ± 0.11* | 9.4 ± 0.74 | 4.94 ± 0.88** | 21.89 ± 1.26 | 26.5 ± 2.86 |
| **S** | 0.86 ± 0.04 | 0.71 ± 0.03 | 9.17 ± 0.35 | 5.86 ± 0.74* | 23.89 ± 1 | 26.31 ± 3.37 |
| **GFP** | 0.79 ± 0.04 | 0.81 ± 0.04 | 7.65 ± 0.66 | 8.24 ± 0.6 | 26.01 ± 2.1 | 27.65 ± 3.36 |
| **Groups** | **Day 45 iPSC-CM data** | | | | | |
| **Ctrl** | 0.7 ± 0.02 | 0.69 ± 0.02 | 8.18 ± 0.37 | 7.06 ± 0.31 | 30.33 ± 1.92 | 23.32 ± 2.09 |
| **E** | 0.67 ± 0.03 | 0.67 ± 0.09 | 5.8 ± 0.31 | 6.24 ± 0.26 | 22.74 ± 2.27 | 26.29 ± 1.87 |
| **M** | 0.74 ± 0.04 | 0.66 ± 0.05 | 6.92 ± 0.57 | 5.68 ± 0.65 | 30.44 ± 3.21 | 19.11 ± 2.28 |
| **N** | 0.72 ± 0.03 | 0.64 ± 0.02 | 6.6 ± 0.61 | 5.61 ± 0.31 | 25.85 ± 3.69 | 22.02 ± 2.23 |
| **ORF-3** | 0.62 ± 0.03 | 0.5 ± 0.05 | 6.36 ± 0.83 | 5.8 ± 0.52 | 26.9 ± 2.95 | 21.28 ± 1.38 |
| **ORF-6** | 0.72 ± 0.04 | 0.54 ± 0.07 | 6.31 ± 0.4 | 6.24 ± 0.52 | 25.48 ± 3.12 | 22.58 ± 1.8 |
| **ORF-7A** | 0.67 ± 0.05 | 0.6 ± 0.06 | 6.91 ± 0.41 | 6.08 ± 0.51 | 26.64 ± 3.18 | 23.93 ± 2.01 |
| **ORF-7B** | 0.71 ± 0.03 | 0.62 ± 0.04 | 6.34 ± 0.45 | 5.09 ± 0.55 | 33.61 ± 3.2 | 19.43 ± 2.13** |
| **ORF-8** | 0.73 ± 0.03 | 0.67 ± 0.05 | 6.46 ± 0.49 | 6.4 ± 0.44 | 28.94 ± 3.14 | 15.06 ± 2.01** |
| **ORF-9A** | 0.71 ± 0.03 | 0.7 ± 0.03 | 6.19 ± 0.57 | 3.98 ± 0.61* | 20.43 ± 2.78 | 21.61 ± 1.7 |
| **ORF-9B** | 0.76 ± 0.03 | 0.69 ± 0.05 | 7.14 ± 0.62 | 4.66 ± 0.31* | 28.63 ± 3.62 | 18.84 ± 2.9 |
| **S** | 0.76 ± 0.05 | 0.58 ± 0.04* | 6.87 ± 0.51 | 4.41 ± 0.49* | 27.84 ± 1.71 | 27.14 ± 2.86 |
| **GFP** | 0.68 ± 0.05 | 0.61 ± 0.05 | 7.23 ± 0.51 | 6.47 ± 0.42 | 30.74 ± 1.39 | 24.63 ± 2.26 |

**Supplemental table 1: Summary of contractility data in iPSC-CMs overexpressed with different COVID-19 proteins**

* and **: p<0.05 and p<0.01 vs non-virus controls from the same group of COVID-19 experiment in 2 way-ANNOVA followed by Sidak’s multiple comparison.
