# Supplementary material for: Ectopic expression of SARS-CoV-2 S and ORF-9B proteins alters metabolic profiles and impairs contractile function in cardiomyocytes
Source: Front Cell Dev Biol. 2023 Feb 22;11:1110271. doi: 10.3389/fcell.2023.1110271 (PMC9994814; doi:10.3389/fcell.2023.1110271)

Supplemental Figure 1

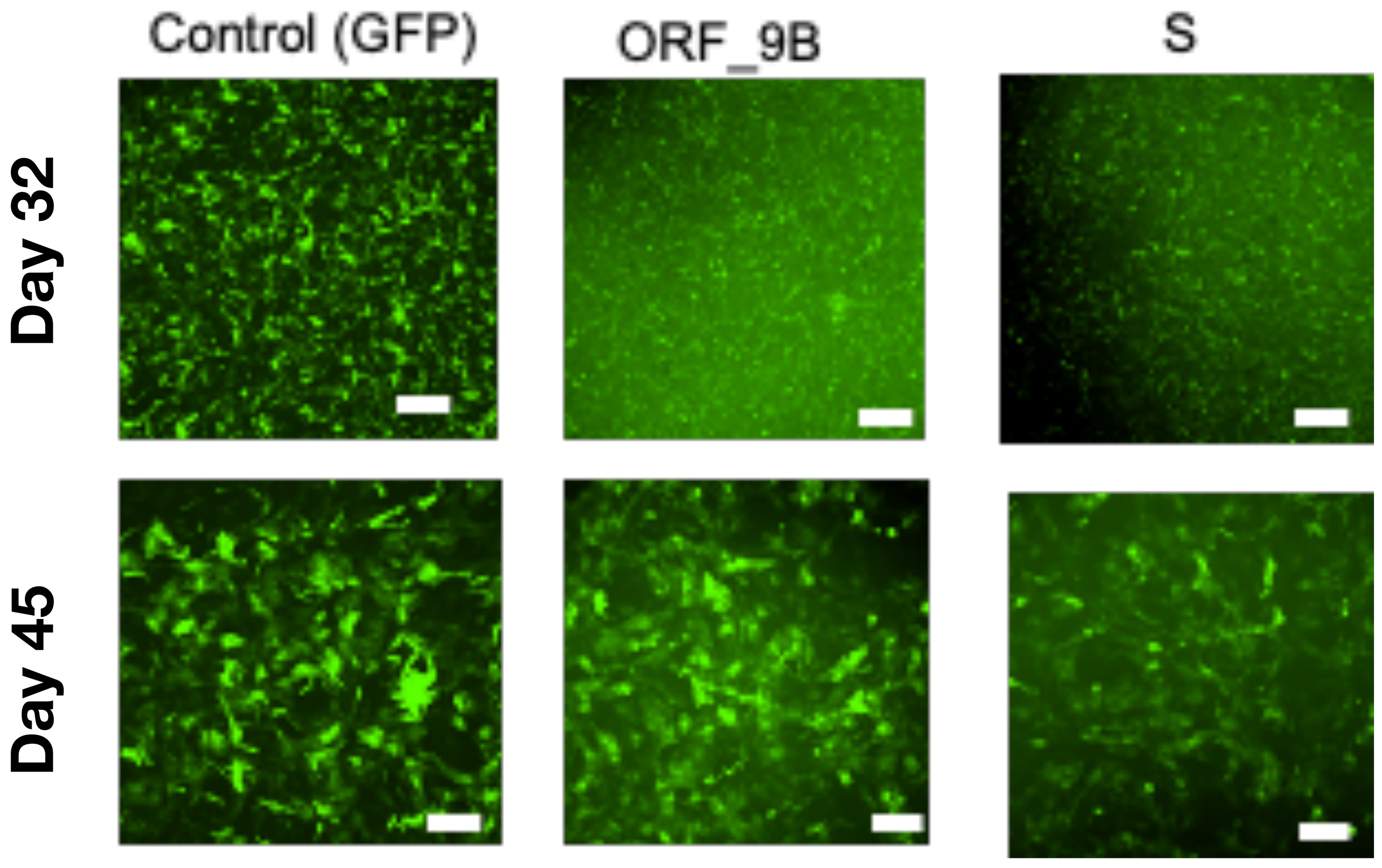

Supplemental Figure 2

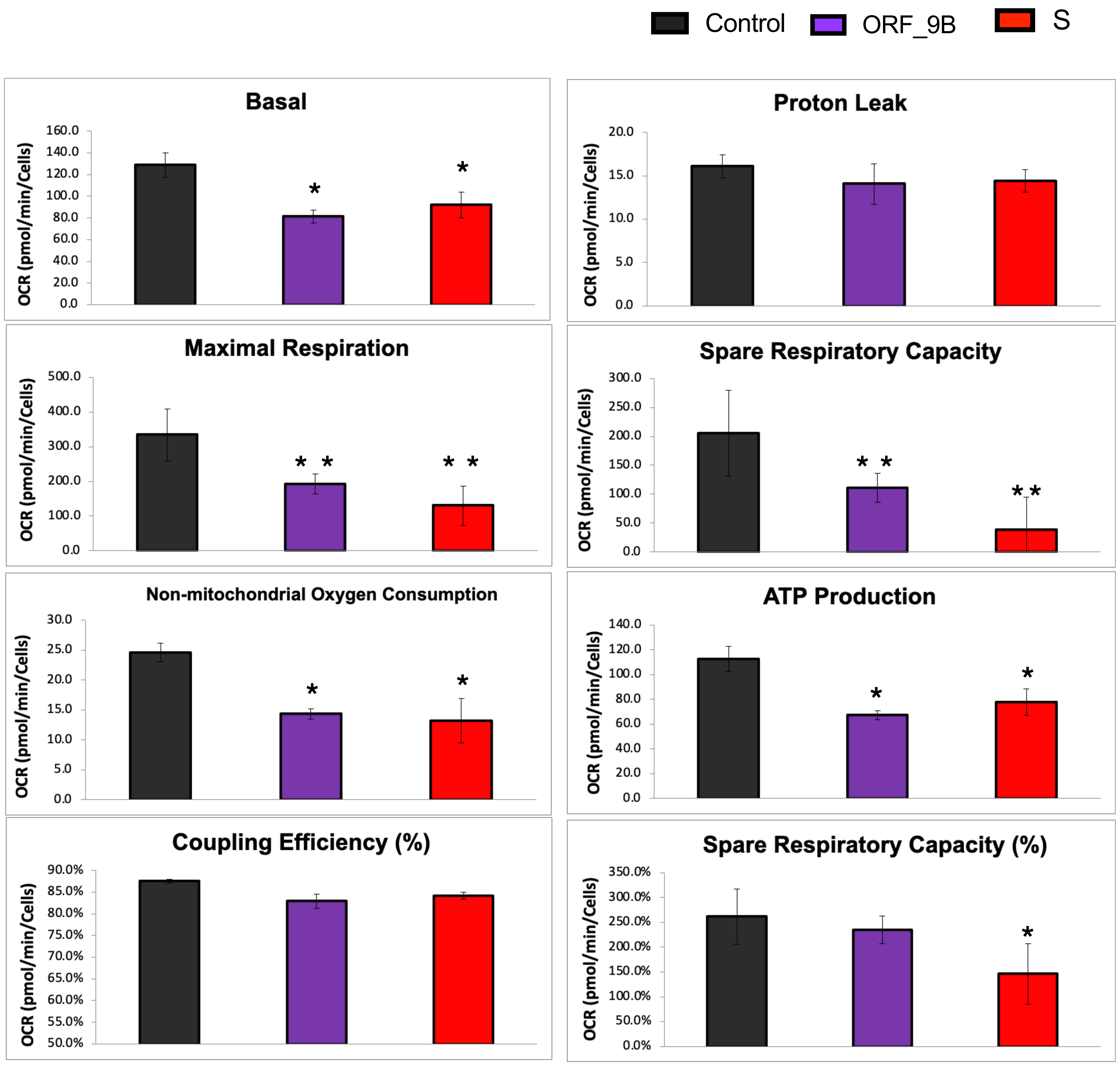

Supplemental Figure 3

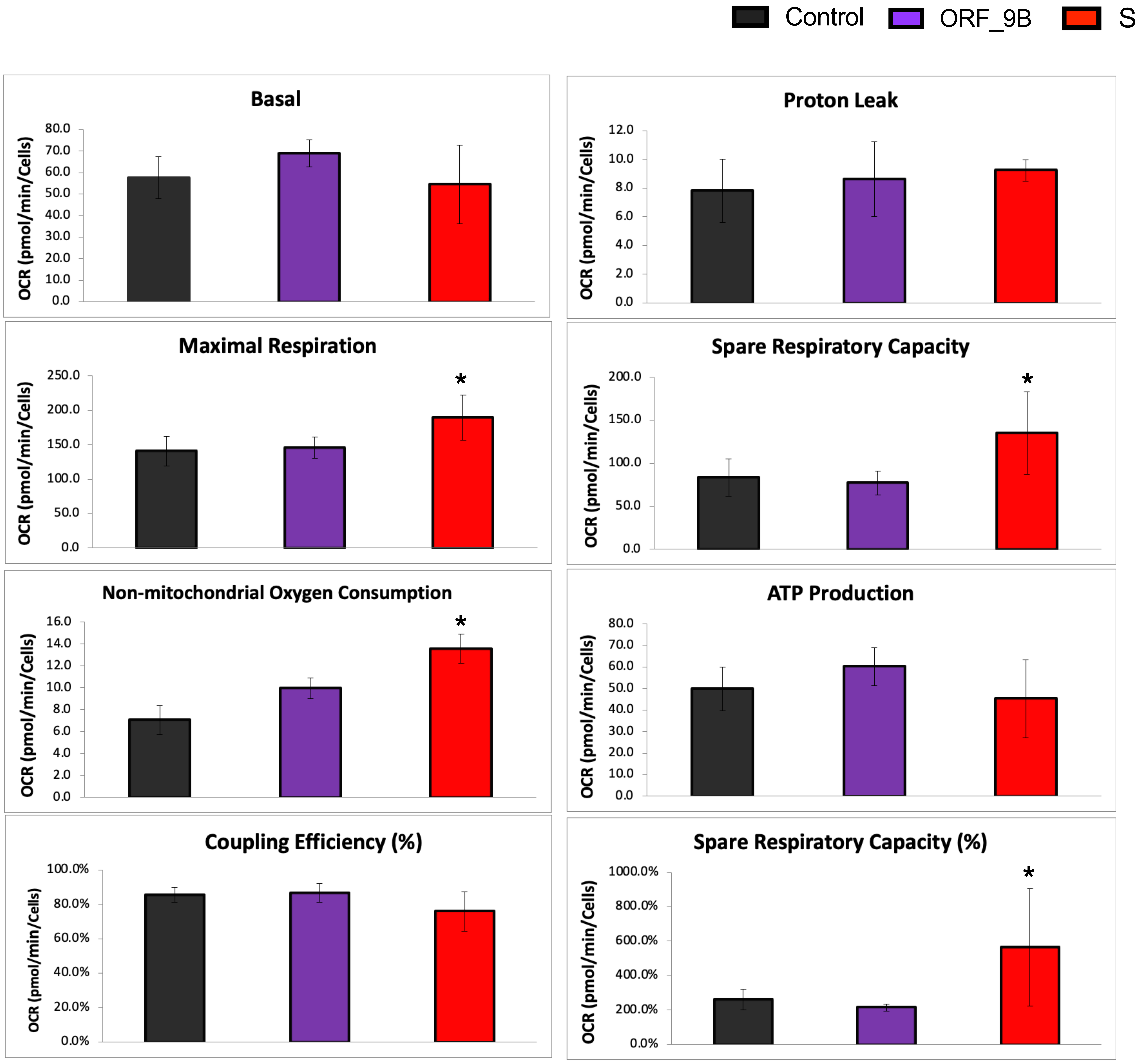

Supplemental Figure 4

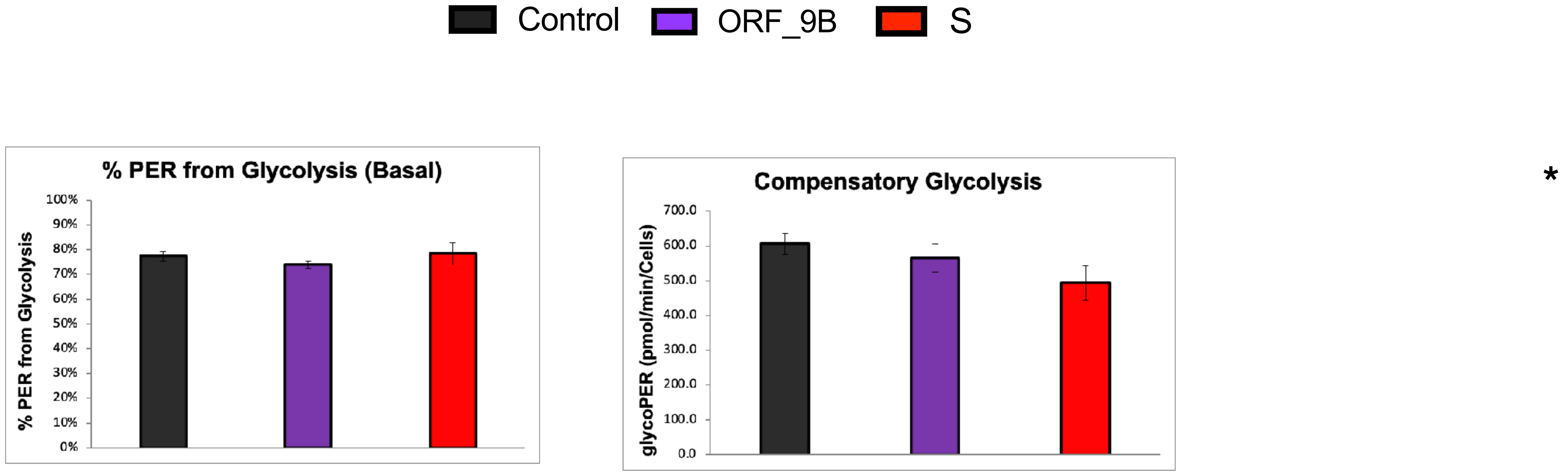

Supplemental Figure 5

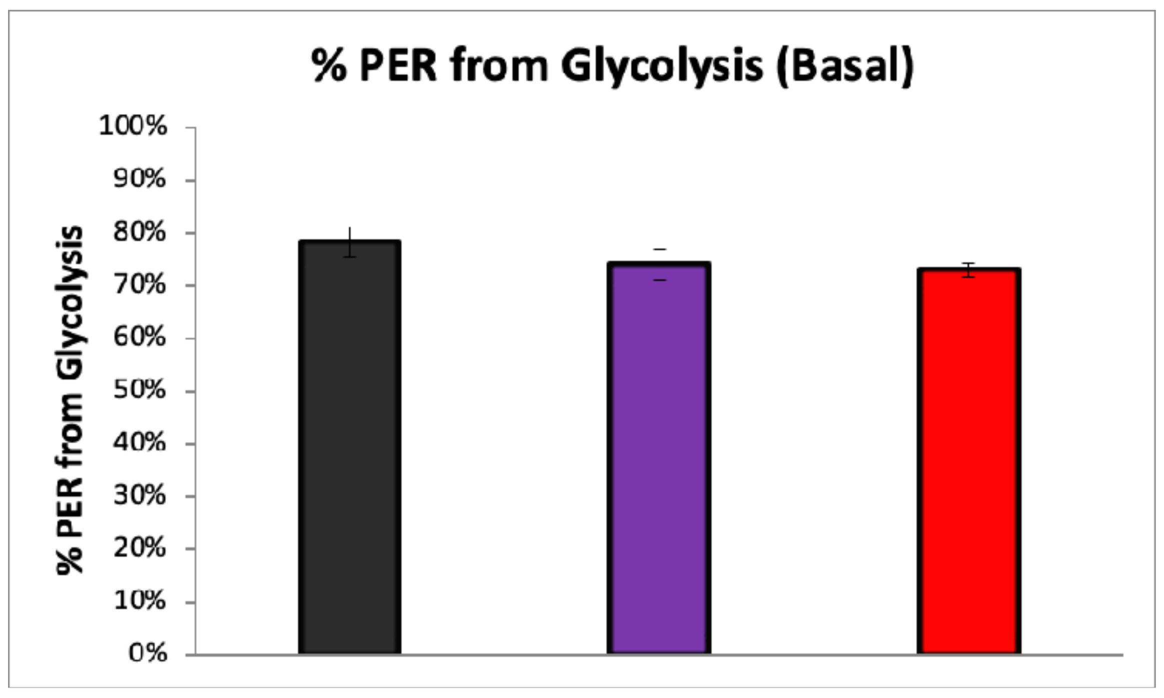

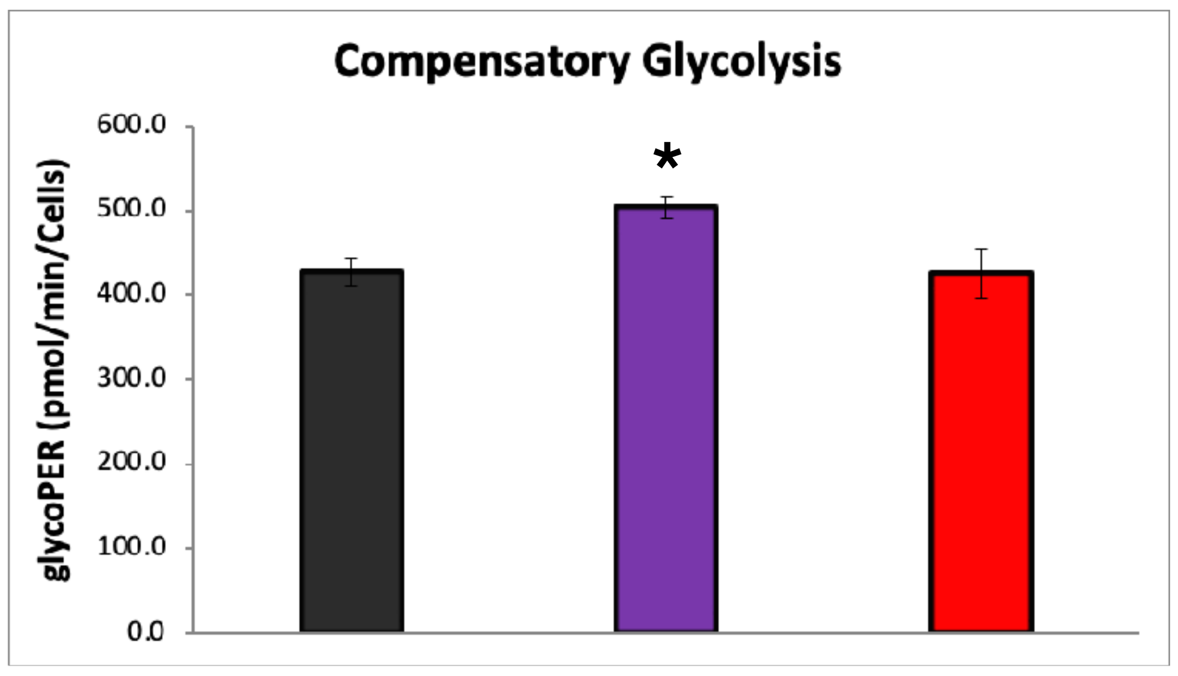

Supplemental Figure 6

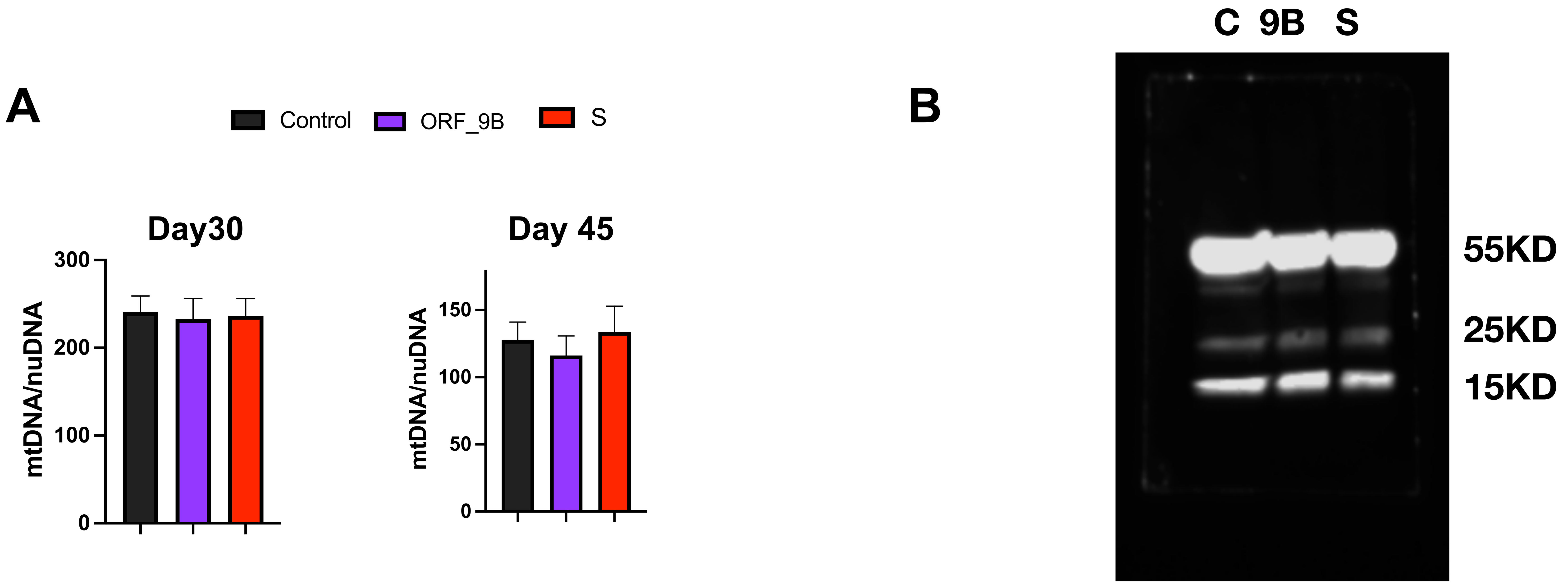

Supplemental Figure 7

A

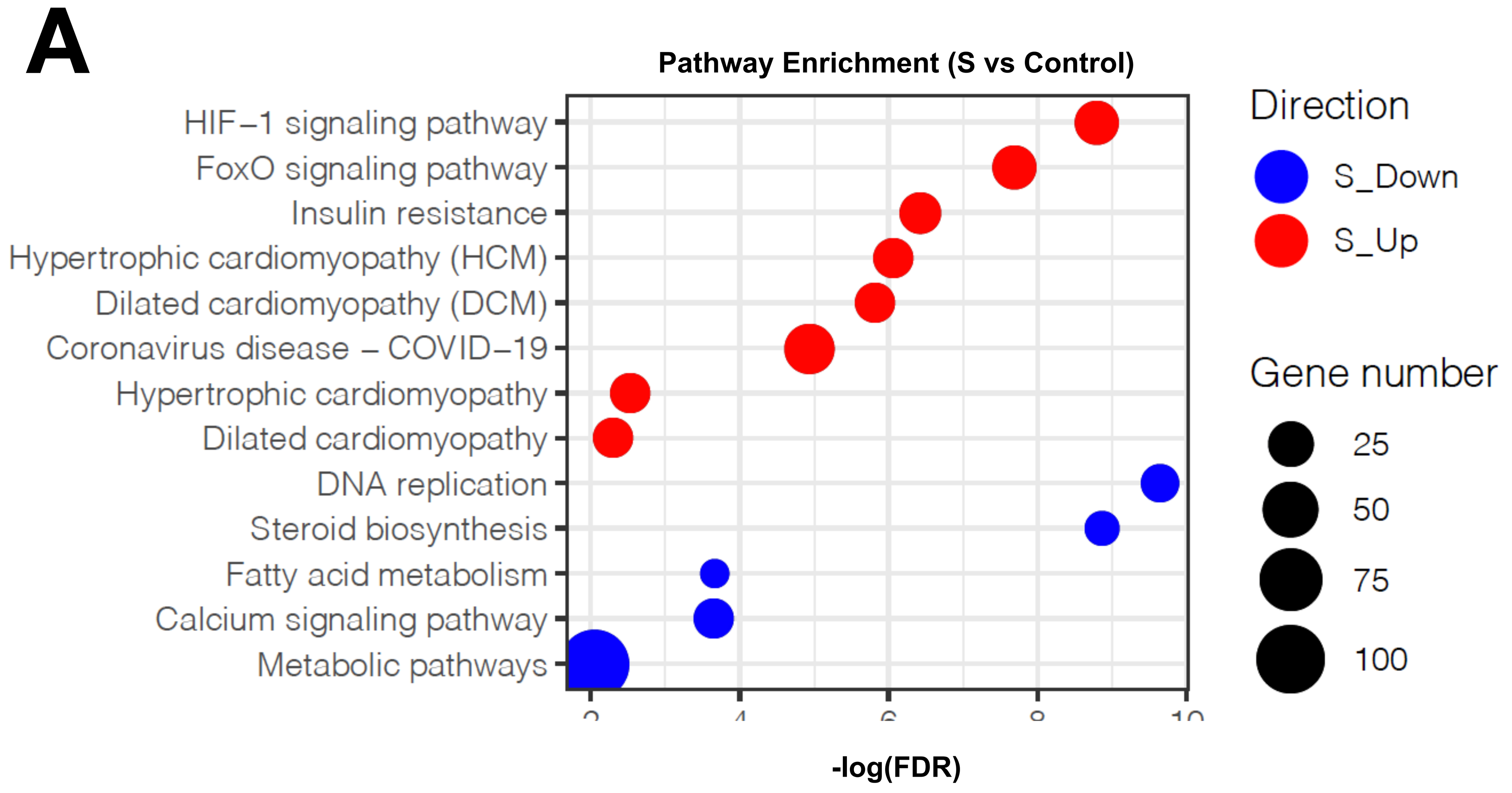

B

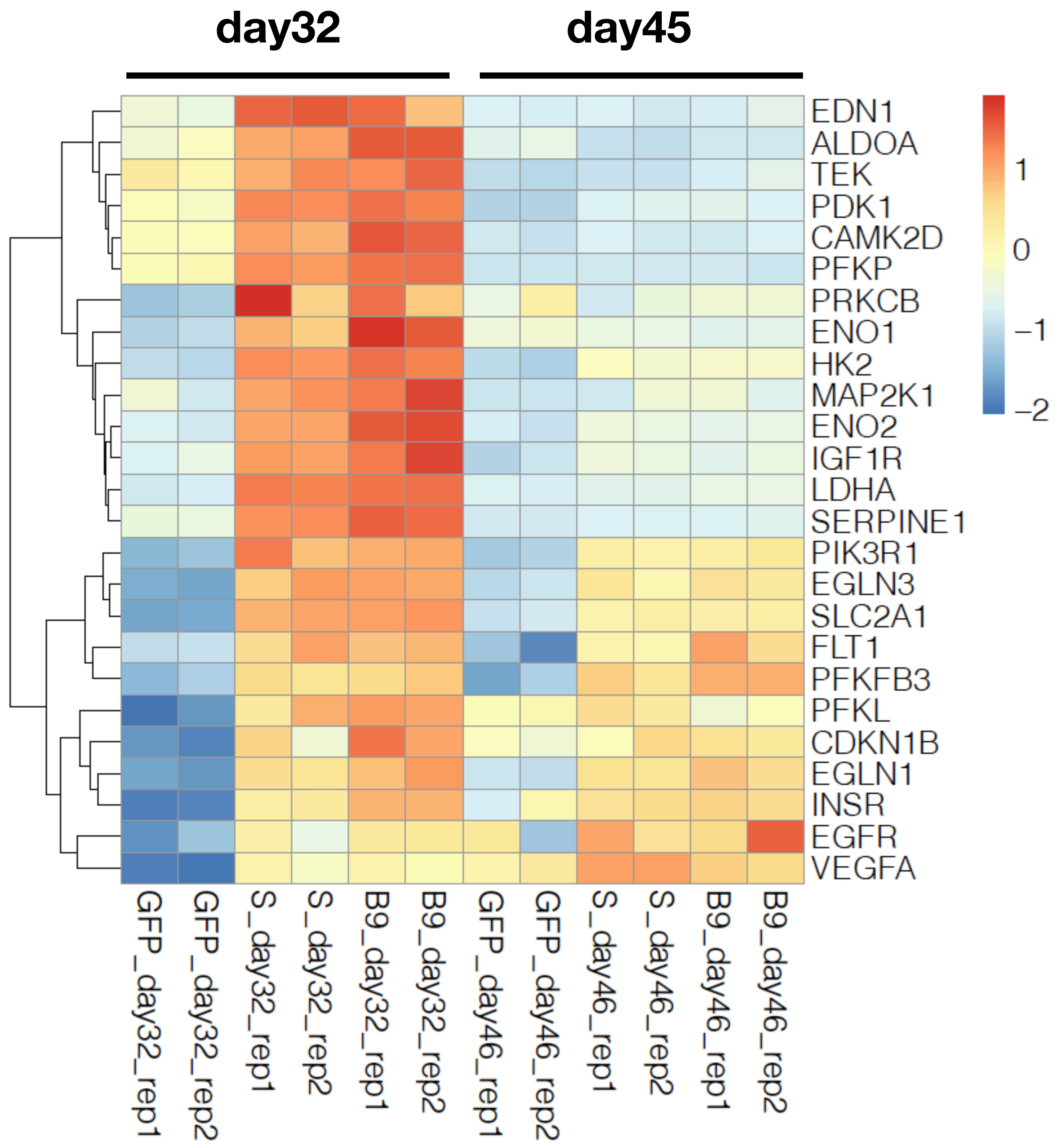

Supplement: Supplementary file 4 [file DataSheet1.PDF]
